# Predictive factors for resection and survival in type A borderline resectable pancreatic ductal adenocarcinoma patients after neoadjuvant therapy: A retrospective cohort study

**DOI:** 10.1097/MD.0000000000032126

**Published:** 2022-12-02

**Authors:** Luis Secanella, Juli Busquets, Núria Peláez, María Sorribas, Berta Laquente, Sandra Ruiz, Thiago Carnaval, Sebastián Videla, Juan Fabregat

**Affiliations:** a Digestive and General Surgery Department, Bellvitge University Hospital, L’Hospitalet DE Llobregat, Barcelona, Spain; b Research Group of Hepato-Biliary and Pancreatic Diseases, Institut d’Investigació Biomèdica de Bellvitge – IDIBELL, University of Barcelona, L’Hospitalet DE Llobregat, Barcelona, Spain; c Departament de Ciències Clíniques, Facultat de Medicina i Ciències de la Salut, Universitat de Barcelona (UB), C. Casanova, Barcelona, Spain; d Medical Oncology Department, Catalan Institute of Oncology, IDIBELL, L’Hospitalet DE Llobregat, Barcelona, Spain; e Radiology Department, Bellvitge University Hospital, L’Hospitalet DE Llobregat, Barcelona, Spain; f Pharmacology Unit, Department of Pathology and Experimental Therapeutics, School of Medicine and Health Sciences, IDIBELL, University of Barcelona, L’Hospitalet DE Llobregat, Barcelona, Spain; g Clinical Research Support Unit (HUB·IDIBELL), Clinical Pharmacology Department, Bellvitge University Hospital, L´Hospitalet DE Llobregat, Barcelona, Spain.

**Keywords:** anatomical definition, borderline resectable, CA 19-9, pancreatic ductal adenocarcinoma

## Abstract

Pancreatic cancer is the seventh leading cause of cancer-related death worldwide, and surgical resection with radical intent remains the only potentially curative treatment option today. However, borderline resectable pancreatic ductal adenocarcinomas (BR-PDAC) stand in the gray area between the resectable and unresectable disease since they are technically resectable but have a high probability of incomplete exeresis. Neoadjuvant treatment (NAT) plays an important role in ensuring resection success.

Different survival prognostic factors for BR-PDAC have been well described, but evidence on the predictive factors associated with resection after NAT is scarce. This study aims to study if CA 19-9 plasmatic levels and the tumor anatomical relationship with neighboring vascular structures are prognostic factors for resection and survival (both Overall Survival and Progression-Free Survival) in patients with type A BR-PDAC.

**Methods:**

This will be a retrospective cohort study using data from type A BR-PDAC patients who received NAT in the Bellvitge University Hospital. The observation period is from January 2010 until December 2019; patients must have a minimum 12-month follow-up. Patients will be classified according to the MD Anderson Cancer Center criteria for BR-PDAC.

**Discussion:**

Patients with BR-PDAC have a high risk for a margin-positive resection. Serum Carbohydrate Antigen 19-9 plasmatic levels and vascular involvement stand out as disease-related prognostic factors.

This study will provide valuable information on the prognostic factors associated with resection. We will exclude locally advanced tumors and expect this approach to provide more realistic resection rates without selecting those patients that undergo surgical exploration. However, focusing on an anatomical definition may limit the results’ generalizability.

## 1. Introduction

### 1.1. Background and rationale

Pancreatic cancer is the seventh leading cause of cancer-related death worldwide, and its incidence and mortality have increased over the last decade.^[1]^ Surgical resection with radical intent remains the only potentially curative treatment option today.^[2]^ Still, only 15% to 20% of pancreatic ductal adenocarcinomas (PDAC) are resectable at diagnosis, while approximately 60% are unresectable due to metastatic disease.^[3]^

Borderline resectable (BR) tumors stand in the gray area between resectable and unresectable disease. They are technically resectable but have a high probability of incomplete exeresis due to their vascular contacts.^[4]^ Currently, chemotherapy ± neoadjuvant chemoradiotherapy is clearly indicated in this subset of patients, making it possible to select those with a potential systemic response that would benefit from surgical resection.

Several BR pancreatic ductal adenocarcinoma (BR-PDAC) definitions have been proposed.^[5]^ Notably, the MD Anderson Cancer Center BR categories^[6]^ were published in 2008 and included 3 patient subsets (types A, B, and C). *Type A (Anatomical*) patients have a BR tumor anatomy (as defined by computerized tomography [CT] imaging) with one or more of the following findings: tumor abutment of the superior mesenteric artery (SMA) or celiac artery (CA) (≤180° of the artery circumference); tumor abutment or encasement of a short segment of the hepatic artery (>180° of the circumference), typically at the origin of the gastroduodenal artery; or short-segment occlusion of the superior mesenteric vein (SMV), portal vein (PV), or SMV-PV confluence that was amenable to vascular resection and reconstruction because of a patent SMV and PV below and above the area of tumor-related occlusion.^[6]^

MD Anderson Cancer Center *Type B (Biological*) patients have BR disease due to a concern for possible extrapancreatic metastatic disease, including those with CT findings suspicious for, but not diagnostic of, metastatic disease and those with known N1 disease.^[6]^ *Type C (Conditional*) patients have BR disease due to marginal or better performance status and severe preexisting medical comorbidity thought to require a protracted evaluation that precluded immediate operation.^[6]^

In 2018, the International Association of Pancreatology (IAP) published a consensus to address possible ambiguous terms used to describe the interface between tumor and vessels in BR-PDAC,^[7]^ IAP Type A patients have either vascular involvement of the SMV and/or PV alone (BR-PV: tumor contact ≥ 180º or invasion of the SMV/PV with bilateral narrowing or occlusion and not exceeding the inferior duodenal border^[7]^) or arterial involvement (BR-A: tumor contact with the SMA and/or CA < 180º without stenosis or deformity; tumor abutment of the common hepatic artery [CHA] without contact with the proper hepatic artery and/or CA^[7]^).

*Type B* patients have tumors potentially resectable anatomically with clinical findings suspicious but not proven distant metastasis (CA 19-9 levels > 500 units/mL, or regional lymph nodes metastasis diagnosed by biopsy or positron emission tomography—Computerized Tomography [PET-CT]). *Type C* patients have anatomically resectable tumors with an Eastern Cooperative Oncology Group ≥ 2.

Currently, the modified FOLFIRINOX or a regimen of Gemcitabine + nanoparticle albumin-bound Paclitaxel are considered the best neoadjuvant treatment (NAT) alternatives for BR-PDACs.^[8]^ The absence of disease progression after NAT is paramount when deciding to indicate the surgery or not.^[9]^

Different survival prognostic factors have been well described for BR-PDACs, but evidence on the predictive factors associated with resection after NAT is scarce. Some authors suggest that the serum carbohydrate antigen 19-9 (CA 19-9) may be a selection criterion for BR-PDAC resection after primary chemotherapy.^[10]^ Similarly, we found that CA 19-9 levels before NAT were lower in the resection group than in the progression group, although not statistically significant.^[11]^ Other authors suggest that a lower Eastern Cooperative Oncology Group performance status and tumor location in the pancreatic neck have higher resection possibilities,^[12]^ whereas BR-PDACs with significant vascular contact have a worse prognosis.^[13–15]^

This study has 2 working hypotheses:

1. In type A BR-PDAC patients who received NAT, CA 19-9 plasmatic levels and the tumor anatomical relationship with neighboring vascular structures are prognostic factors for resection.
2. In type A BR-PDAC patients who received NAT, CA 19-9 plasmatic levels and the tumor anatomical relationship with neighboring vascular structures are prognostic factors for survival (both Overall Survival and Progression-Free Survival).

### 1.2. Objectives

Our main objectives will be:

1. To estimate the rate of patients who started NAT (≥3 cycles) and underwent surgical exploration and resection.
2. To study the predictive factors associated with resection in type A BR-PDAC patients who received NAT.
3. To study the survival and disease progression in type A BR-PDAC patients who started NAT.
4. To study the predictive factors associated with survival of type A BR-PDAC patients who received NAT.

Our *secondary objectives* will be:

1. To perform an exploratory comparative analysis between both groups of patients (resected vs non-resected).
2. To estimate how many patients who received NAT (by the number of cycles) present death, disease progression, stable disease, or response at the end of the observation period.

We will consider *disease progression* as the development of metastatic disease and/or an increase in the primary tumor size. We will consider *response* when the primary tumor presents a reduction in size and/or in its relationship with neighboring vascular structures. On the other hand, we will consider as *stable disease* an insufficient increase or reduction in the primary tumor size or in its relationship with neighboring vascular structures (i.e., cases that cannot be classified as responders).

## 2. Methods

### 2.1. Study design

**Figure 1.**
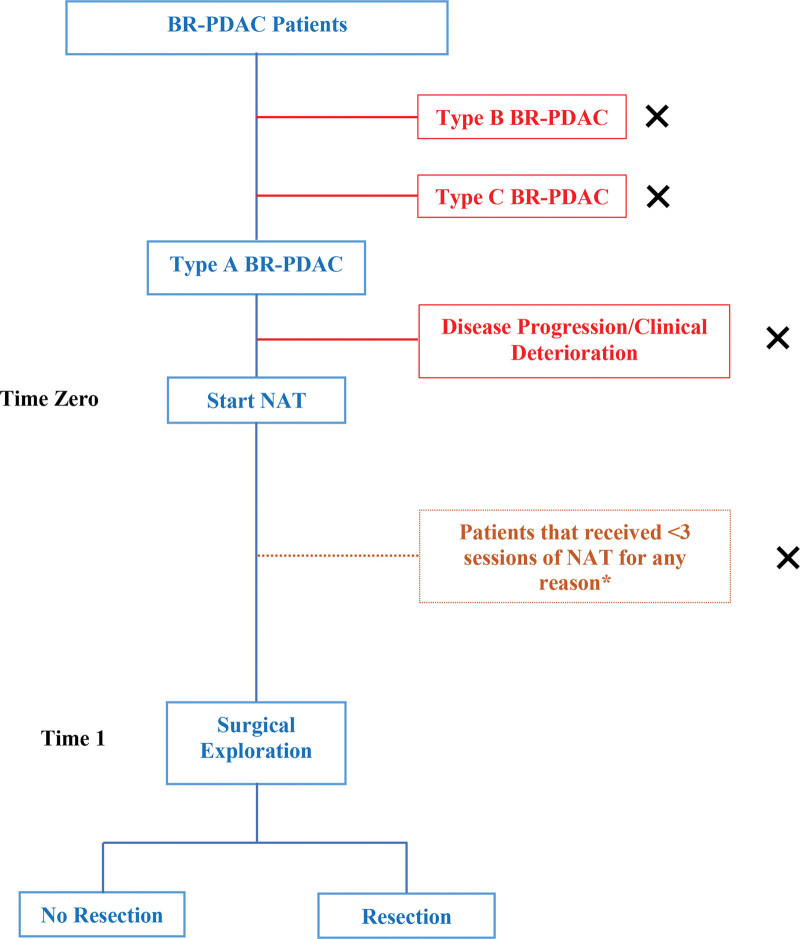
Flowchart. BR-PDAC = borderline resectable pancreatic ductal adenocarcinoma, NAT = neoadjuvant treatment, X = Excluded. *Patients who received < 3 NAT cycles will only be excluded from the surgical exploration analysis.

This will be a retrospective cohort study using data from type A BR-PDAC patients who received NAT in the Bellvitge University Hospital (BUH). The observation period is from January 2010 until December 2019; patients must have a minimum 12-month follow-up. A flowchart summarizing the study design is shown in Figure 1.

Given the initial disparity of definitions prior to the introduction of the IAP criteria in 2018, the retrospective nature of this study, and the fact that our Department used the MD Anderson Cancer Center criteria until 2017, we will follow the criteria set by the MD Anderson Cancer Center to classify BR-PDACs. For the purpose of this study, we will only consider type A BR-PDAC.

The start of the NAT will be “Time Zero.” “Time 1” will be the moment of surgical exploration, when patients will be stratified into those who underwent resection and those who did not (resected and non-resected). Patients will be followed-up until death or until the last medical control registered in the electronic medical file (censored).

### 2.2. Staging

We will gather demographic and clinical data from each included patient. Baseline radiological staging was performed in all patients per usual clinical practice using a 4-phase multi-detector CT scan—64 (64-MDCT). Additional examinations such as hepatic magnetic resonance imaging or ^18^FDG PET-CT were performed when the information from the 64-MDCT was insufficient to classify the PDAC properly.

As mentioned before, we will follow the criteria set by the MD Anderson Cancer Center to classify BR-PDACs. Therefore, for pancreatic head tumors, any contact <180º with the SMA or CHA or ≥180º with the SMV or PV (including their narrowing/occlusion when surgical reconstruction was feasible) is considered a BR-PDAC. For pancreatic neck/body tumors, any contact with the CA or CHA is regarded as a BR-PDAC.

All cases were discussed in the institutional Multidisciplinary Pancreas Committee. Patients ultimately diagnosed with a BR-PDAC underwent biliary drainage (in case of jaundice) and cytological confirmation by ultrasonography-guided endoscopy.

### 2.3. Neoadjuvant treatment

Currently, NAT consists of Gemcitabine-based or FOLFIRINOX based regimens. From 2010 to 2017, NAT was based on the Gemcitabine + Oxyplatin regimen for 6 cycles. In 2017, Gemcitabine + Oxyplatin regimen was replaced by the Gemcitabine + nanoparticle albumin-bound Paclitaxel regimen, also for 6 cycles. After the 6^th^ chemotherapy cycle, a 64-MDCT is performed for restaging.

Additionally, some patients without evidence of disease progression received 5-Fluorouracil with concomitant radiotherapy. Only patients with excellent functional class received the modified FOLFIRINOX regimen (5-Fluorouracil, Leucovorin, Oxaliplatin, and Irinotecan).

It is worth mentioning that our study group took part in 2 clinical trials using specifically neoadjuvant chemotherapy regimens (GEMERLOXA^[11]^ and NALIRINOX [NCT04010552]) during the observation period. Thus, the target population will have undergone different chemotherapy regimens, although all have been administered in a neoadjuvant context.

Once the NAT was concluded, patients underwent a new 64-MDCT scan to assess tumor response. Those without disease progression were selected for surgical resection. The minimum time interval between the last NAT session and the resection was 4 weeks.

### 2.4. Radiological response

As mentioned before, all patients underwent a new 64-MDCT scan for restaging after NAT. This is the most frequently used imaging method to evaluate PDAC response after NAT^[16]^ due to its higher spatial resolution and multiplanar reconstruction ability.^[16,17]^ However, it also has limitations such as a relatively low sensitivity in assessing the resectability after NAT and low specificity in predicting R0 resections due to its inaccurateness in distinguishing between residual tumor and tissue scarring after tumor regression.^[16]^

The Response Evaluation Criteria in Solid Tumors (RECIST—version 1.1)^[18]^ have been commonly used to assess treatment response. However, some authors pointed out that the RECIST criteria are not suitable for BR-PDACs response assessment after NAT since these tumors present few morphological changes after treatment.^[16,19]^ Therefore, we will classify the NAT outcome as *response*, *stable disease*, or *disease progression* as previously explained in this manuscript. Patients without disease progression were selected for surgical exploration.

### 2.5. Tumor biomarker

CA 19-9 is a dynamic preoperative tumor biomarker and a useful tool to assess NAT responsiveness. It also provides prognostic information in resected and non-resected BR-PDAC patients.^[20]^

As per usual clinical practice, we measure the CA 19-9 plasmatic level before the 64-MDCT scan, after biliary drainage (in those who need it), and after the NAT.

Patients with non-secreting tumors have baseline CA 19-9 plasmatic levels < 37 U/mL. At the end of NAT, we reassess the CA 19-9 plasmatic levels and compare them to the pretreatment levels. Biochemical responders have reductions >10% from baseline; biochemical normalization is achieved when CA 19-9 < 37 U/mL.

### 2.6. Settings

This study will be carried out at the BUH by its medical staff members (Digestive and General Surgery Department, Radiology Department, and Clinical Pharmacology Department).

### 2.7. Participants

All BR-PDAC patients evaluated by the Digestive and General Surgery Department (Hepatobiliary and Pancreatic Surgery Unit) from January 2010 to December 2019 will be assessed for eligibility.

#### 2.7.1. Inclusion criteria

Patients ≥18 years old, of both sexes, diagnosed with a type A BR-PDAC between January 2010 and December 2019, and with a minimum follow-up period of 12 months.

#### 2.7.2. Exclusion criteria

Patients diagnosed with a type B or type C BR-PDAC; patients diagnosed with a type A BR-PDAC who had disease progression prior to receiving NAT.

### 2.8. Variables

Our *main variables* will be:

1. The *number* of type A BR-PDAC patients who, after receiving NAT (≥3 cycles), undergo *resection*.
2. The *evolution* of the plasmatic levels of *CA 19-9* from “Time Zero” until “Time 1.”
3. The *evolution* of the *degree of vascular involvement* from “Time Zero” until “Time 1.”
4. Overall Survival: *time until death* (for any cause) from “Time Zero” until the end of the observation period.
5. The *evolution* of the plasmatic levels of *CA 19-9* from “Time Zero” until death.
6. The *evolution* of the *degree of vascular involvement* from “Time Zero” until death.
7. Progression-Free Survival: *time until disease progression* from “Time Zero” until the end of the observation period.
8. The *evolution* of the plasmatic levels of *CA 19-9* from “Time Zero” until Progression-Free Survival.
9. The *evolution* of the *degree of vascular involvement* from “Time Zero” until Progression-Free Survival.

Our *secondary variables* will be:

1. The n*umber* (percentage) of deaths at the end of the observation period.
2. *The number (percentage*) of patients presenting *disease progression* at the end of the observation period.
3. *The number (percentage*) of patients presenting *stable disease* at the end of the observation period.
4. *The number (percentage*) of patients presenting *response* at the end of the observation period.
5. *The number (percentage*) of patients *surgically explored* at the end of the observation period.
6. *The Resection Rate* at the end of the observation period.

The Resection Rate will be calculated by dividing the total number of resections performed by the total number of patients treated with NAT.

### 2.9. Data source and ethical issues

The study data source will be the patients’ individual electronic medical records. Data will be collected retrospectively.

We will ensure data quality by recording all study-related participant data in a unified, *ad-hoc*-created dataset. The investigators will be responsible for verifying data entries’ accuracy and correctness. Moreover, the sponsor (or its designee) will be responsible for the data management and overseeing data quality.

The study protocol (version 1.1) received local Institutional Review Board approval (IRB) on July 1^st^, 2022 (Ethics and Clinical Investigation Committee of the BUH, code EOM023/22). The list of local IRB members is available at: https://bellvitgehospital.cat/es/investiga-con-nosotros/ceic/composicion (accessed on July 20^th^, 2022).

Investigators will comply with any data monitoring procedures related to IRB or any other competent authority audits and will provide direct access to the study’s master files.

This study will be conducted under the criteria set by the Declaration of Helsinki (revised on WMA 64^th^ General Assembly, Fortaleza, Brazil, October 2013), good clinical practice standards, and applicable regulations. The level of confidentiality protection, in terms of personal data protection, as required by Spanish Law (Organic Law on Data Protection 3/2018), will also be ensured.

All data collected during the study will undergo dissociation as Spanish law requires. The datasets will be pseudonymized by a member of the Clinical Research Support Unit (independent from the study). Patients will be identified in the datasets with an ad-hoc-created code to ensure anonymization. The study team commits not to carry out any activity to re-identify the patients once the dataset has been closed for analysis and to adopt specific security measures to prevent re-identification and access by unauthorized third parties. Investigators’ participation in this study is free, voluntary, unpaid, and independent.

The datasets generated and/or analyzed during the current study will be available from the corresponding author on reasonable request.

### 2.10. Bias

Despite the retrospective study design, datasets have been designed *ad-hoc*, even though the data source will be the individual electronic medical records. We established a minimum 12-month follow-up completeness as an inclusion criterion to ensure enough time for the outcomes to occur. Besides, considering that many endpoints are hard variables or standard clinical practice laboratory data, we do not expect any issues when fulfilling the datasets.

Although we based our diagnostic criteria on updated evidence and kept diagnostic homogeneity among study group members by following our established standard clinical practice guidelines, changes in these diagnostic criteria were inevitable in a 10-year observation period. BR-PDAC definitions became wider throughout the years (both for tumor anatomy and patients’ performance status), which might contribute to selection bias.

Also, different NAT regimens have been used. We will perform a multivariate analysis to assess their impact as a prognostic factor. This may address this issue properly.

### 2.11. Study size

Given the study’s exploratory nature, a formal sample size calculation has not been carried out. All patients who meet all the inclusion criteria and none of the exclusion criteria will be included. We expect to include approximately 100 patients.

### 2.12. Statistical methods

We will perform a general descriptive analysis of all study variables. The results will be expressed as means (standard deviation [SD]) or medians (range) for the quantitative variables and as absolute and relative frequencies for the categorical variables.

We will use Pearson’s *χ*^2^ (or Fisher’s exact test, when appropriate) to perform a comparative analysis of the categorical variables. For the continuous variables, we will use the Student *t* test (if the distribution is normal) or the Mann–Whitney U for the non-parametric variables. Overall Survival and Progression-Free Survival will be estimated using the Kaplan–Meier model and compared using the Log-Rank test. Unless stated otherwise, two-sided 95% confidence intervals will be reported. The timeline for the survival analysis will be from the start of the NAT until December 2020 and the events of interest will be death or disease progression.

Since different NAT regimens have been used, we will perform a multivariate analysis to assess their impact as a prognostic factor.

The statistical analysis will be performed with Stata® Software 12.0 (Stata Corp LP, Lakeway Drive, TX) or higher.

## 3. Discussion

BR-PDAC patients have a high risk for a margin-positive resection, and many study groups have tried to define BR-PDACs accurately^[7]^ since these patients could benefit from NAT. Much effort has been put into finding objective criteria for stage-specific therapy and creating reproducible eligibility criteria for clinical trials.^[6]^ In this scenario, 2 disease prognostic factors stand out: the plasmatic levels of CA 19-9 and the vascular involvement.

The prognostic value of the CA 19-9 at the end of the NAT is currently well established, but only a handful of studies focused on the CA 19-9 relationship with resectability. A reduction in CA 19-9 plasmatic levels >10% (compared to baseline levels) are deemed a favorable response to NAT, and CA 19-9 response to NAT is a better indicator of the tumor’s biological behavior than its absolute pretreatment values.^[8,9,12,20]^

Vascular involvement is also widely accepted as a prognostic factor for BR-PDACs. CHA/CA involvement at diagnosis suggests a poorer outcome. Some authors reported significantly lower survival rates in patients with arterial involvement compared to SMV/PV involvement^[14]^; others found arterial abutment and SMV/PV encasement to be independent prognostic factors.^[15]^ Accordingly, a better prognosis was reported when radiographic signs of CHA involvement were absent.^[13]^

However, the available evidence on the prognostic factors for resection in BR-PDAC patients is scarce, and the majority of studies conducted until now calculate their resection rates over the number of patients that undergo exploratory surgery. We believe this study will provide valuable information on these prognostic factors associated with resection and, unlike previous studies, it will obtain the resection rate by dividing the total number of resections performed by the total number of patients who received ≥ 3 cycles of NAT. Additionally, we will exclude all locally advanced PDACs because other unknown factors could play a specific role in their evolution and management. We expect this approach to provide more realistic resection rates without selecting those patients that undergo surgical exploration.

This study may have limitations. Besides what was mentioned in the Bias section, focusing on an anatomical definition may limit the generalizability of the results because it does not consider the tumor biology or the host’s physiology, both determinants of resectability.^[7]^ Another issue may be the known worst prognosis of Lewis-negative PDACs,^[21]^ which is not possible to identify in our setting; the alternative DUPAN-2 biomarker is also unavailable in our setting. Therefore, the only tumor biomarker we will use will be the CA 19-9, and patients will be classified as having secreting or non-secreting tumors.

*This manuscript complies with the STROBE statement.* ^[22]^

### 3.1. Generalizability

This study will be conducted using real-word data.

## Acknowledgments

We thank Bellvitge University Hospital, IDIBELL and CERCA Program/Generalitat de Catalunya for institutional support.

## Author contributions

**Conceptualization:** Luis Secanella, Juli Busquets, Thiago Carnaval, Sebastián Videla, Juan Fabregat.

**Funding acquisition:** Juli Busquets, Juan Fabregat.

**Investigation:** Luis Secanella, Juli Busquets, Núria Peláez, María Sorribas, Berta Laquente, Sandra Ruiz, Juan Fabregat.

**Methodology:** Juli Busquets, Thiago Carnaval, Sebastián Videla.

**Supervision:** Luis Secanella, Juli Busquets, Sebastián Videla, Juan Fabregat.

**Writing – original draft:** Luis Secanella, Juli Busquets, Thiago Carnaval, Sebastián Videla.

**Writing – review & editing:** Luis Secanella, Juli Busquets, Berta Laquente, Sandra Ruiz, Thiago Carnaval, Sebastián Videla.
